# Supplementary material for: Limited inhibition of multiple nodes in a driver network blocks metastasis
Source: eLife. 2021 May 11;10:e59696. doi: 10.7554/eLife.59696 (PMC8128439; doi:10.7554/eLife.59696)

Figure 2 - source data 2

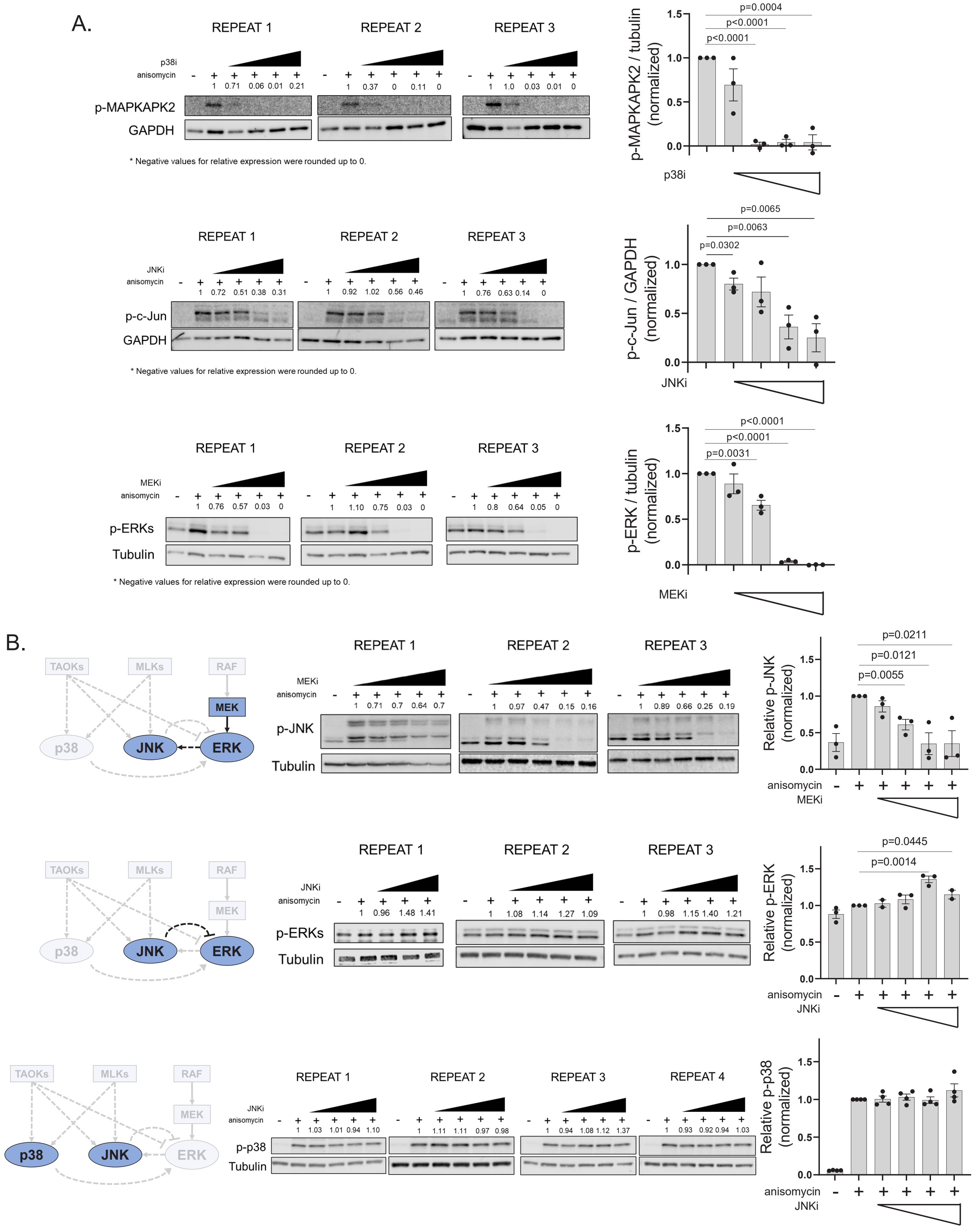

Figure 2 - source data 2 (cont'd)

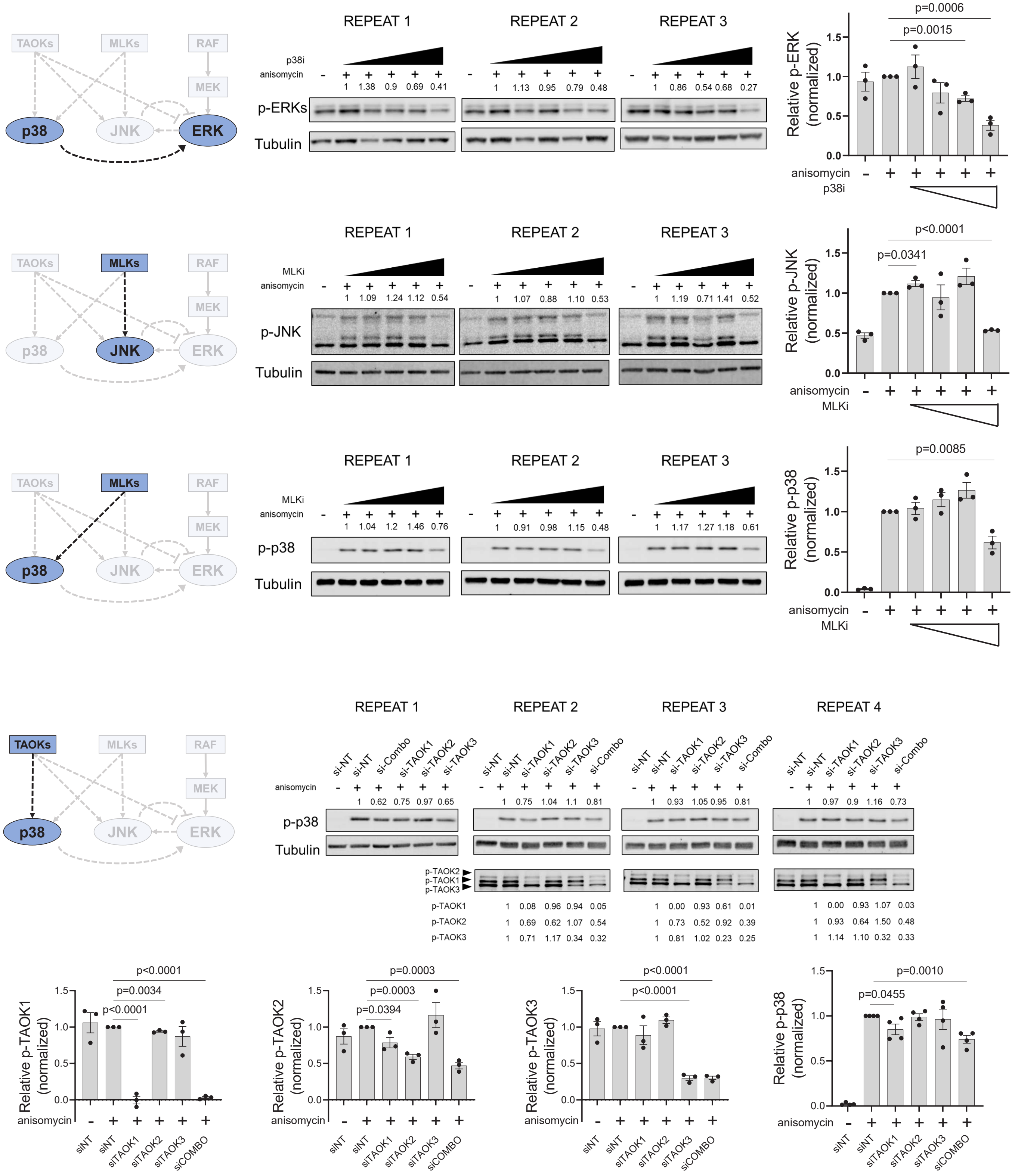

Figure 2 - source data 2 (cont'd)

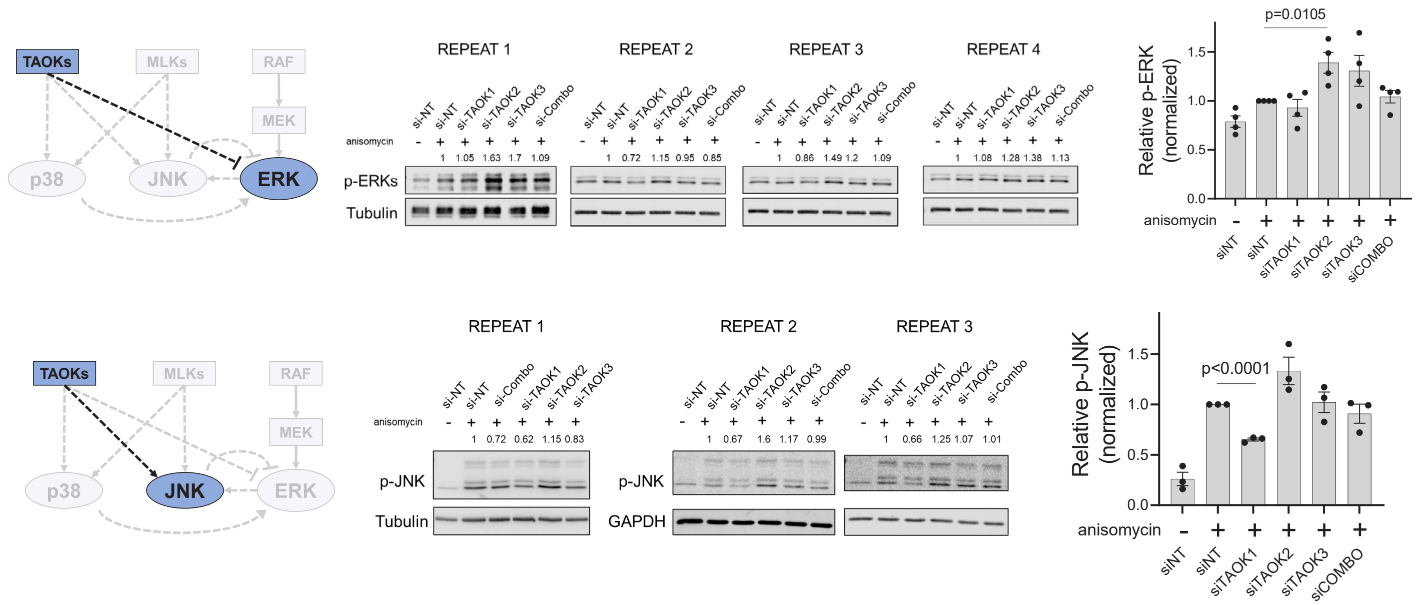

Supplement: Figure 2—source data 2. — (A) Western blots from three independent experiments showing the effect of p38i, JNKi, and MEKi on the phosphorylation of known direct targets of p38, JNK, and MEK, respectively. To monitor the substrate phosphorylation of p38, JNK, and MEK under treatment, we chose their known substrates MAPKAPK2, c-Jun, and ERK1/2, respectively. Statistical test was performed by student’s t-test for each dose of the inhibitors with respect to the non-treated control sample. (B) Western blots from three or more independent experiments showing the crosstalk and feedbacks that exist within the BM1 stress-kinase MAPK network. The diagrams on the far left show the specific direct/indirect interaction between two kinase nodes of the network, determined by inhibition of one of the kinases with a small molecule inhibitor or siRNAs and monitoring the activity of the other kinase. For this analysis, we used canonical phosphorylation sites of each kinase that correlate with their activity based on literature. The results of these experiments were used to build the network topology for BM1 cells depicted in Figure 2F. [file elife-59696-fig2-data2.pdf]
