# Supplementary material for: Limited inhibition of multiple nodes in a driver network blocks metastasis
Source: eLife. 2021 May 11;10:e59696. doi: 10.7554/eLife.59696 (PMC8128439; doi:10.7554/eLife.59696)

Assay 1 - Single drug dose-response

Conditions tested:

1-EGF (positive control with chemoattractant, but no drug)
2-no-EGF (negative control for baseline invasion, no chemoattractant, no drug)
3-p38i (1μM, 10μM, 25μM)
4-JNKi (1μM, 10μM, 25μM)
5-MEKi (1nM, 10nM, 100nM, 1μM)
6-MLKi (10nM, 200nM, 1μM, 10μM)
7-TAOK2i (0.1μM, 1μM, 10μM, 25μM)
8-CK2i (0.1μM, 1μM, 10μM, 25μM)

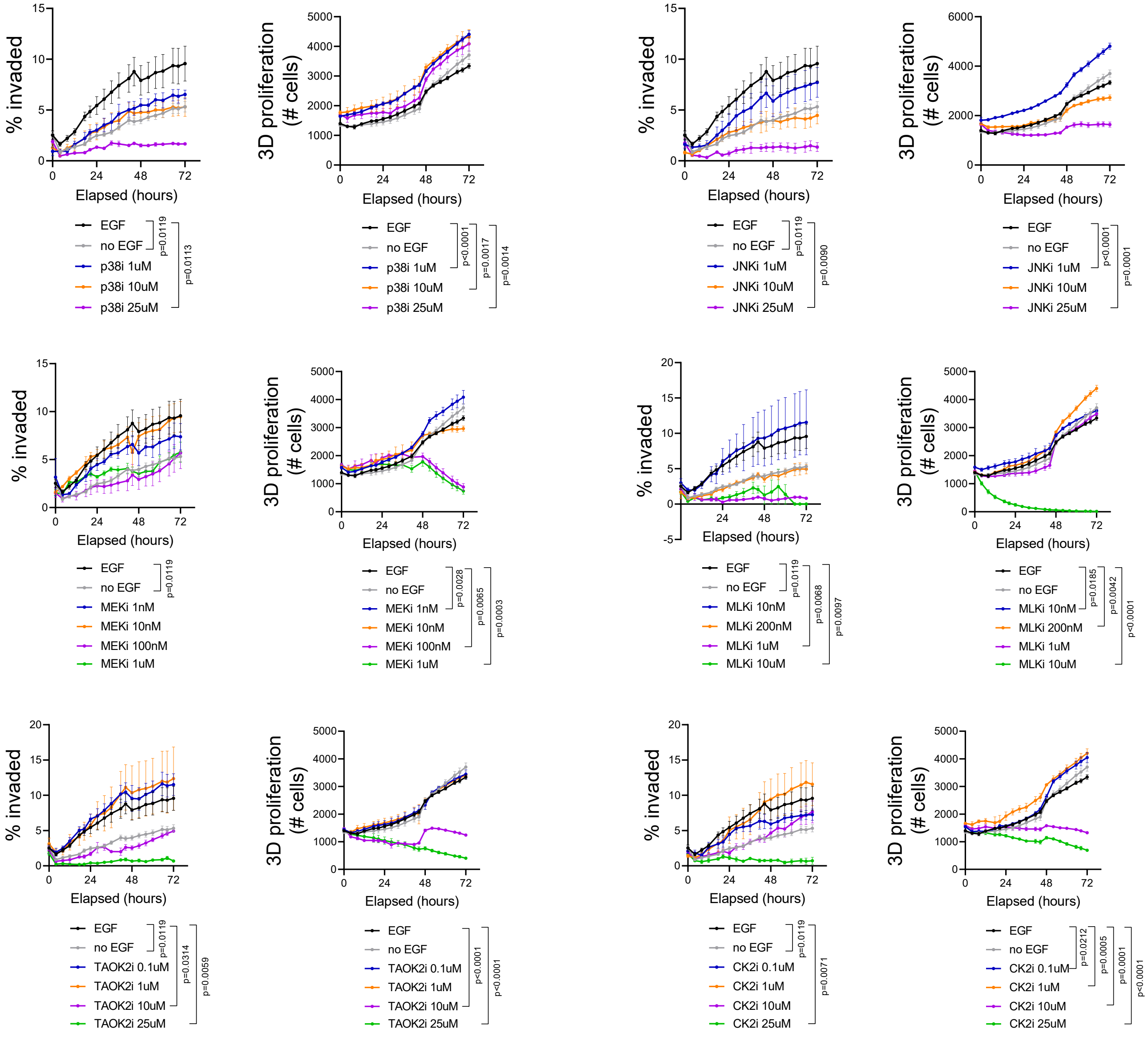

Assay 2 - Dual drug combinations (1)

Conditions tested:

1-EGF (positive control with chemoattractant, but no drug)
2-no-EGF (negative control for baseline invasion, no chemo-attractant, no drug)
3-JNKi (1μM)
4-p38i (1μM)
5-MEKi (10nM)
6-MLKi (10nM)
7-CK2i (0.1μM)
8-TAOK2i (1μM)
9-JNKi + CK2i
10-JNKi + MEKi
11-JNKi + MLKi
12-JNKi + p38i
13-JNKi + TAOK2i
14-MEKi + CK2i
15-p38i + CK2i
16-p38i + MEKi
17-p38i + MLKi
18-p38i + TAOK2i
19-TAOK2i + MEKi
20- TAOK2i + MLKi

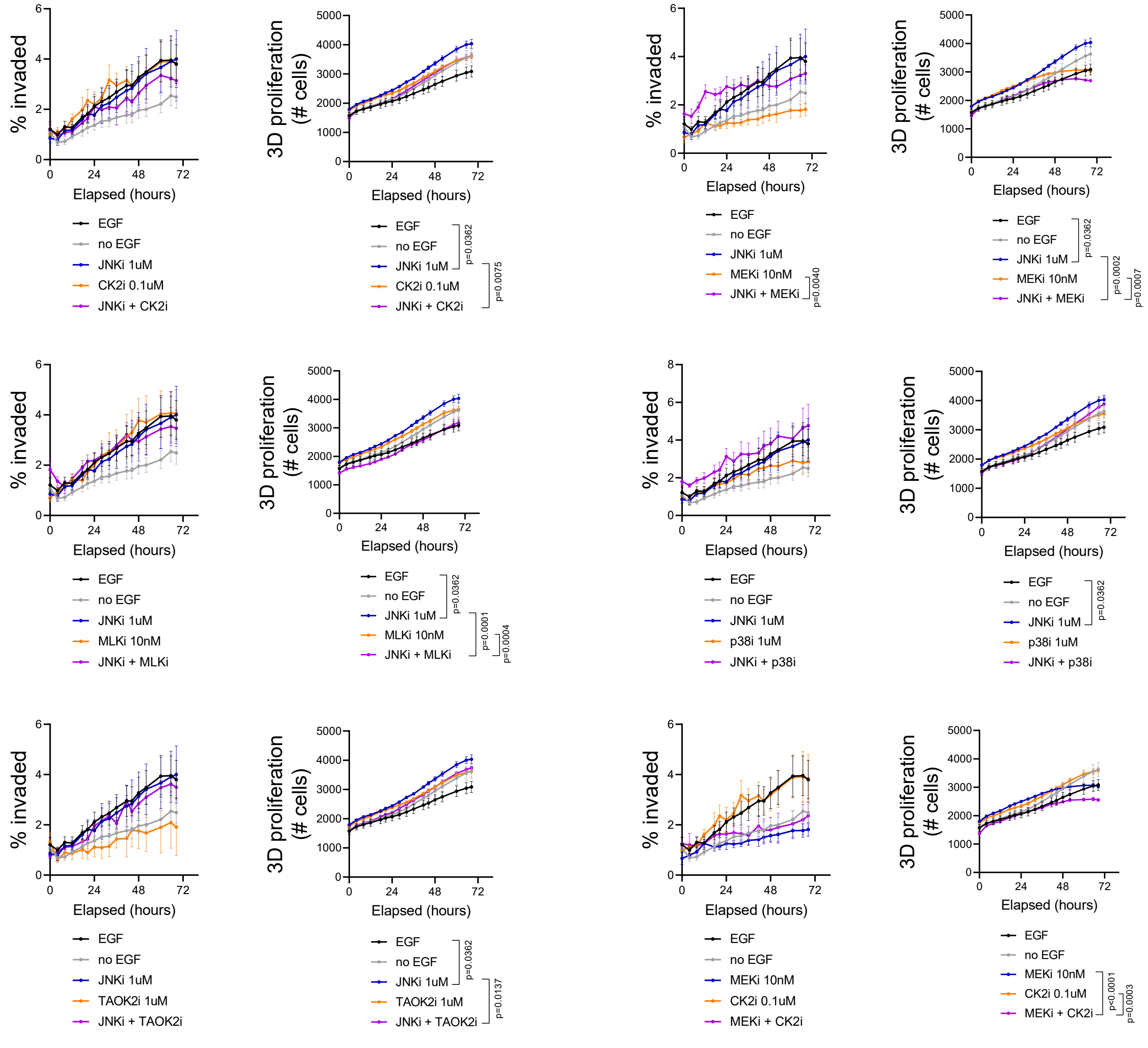

Figure 3 - source data 3 (cont'd)

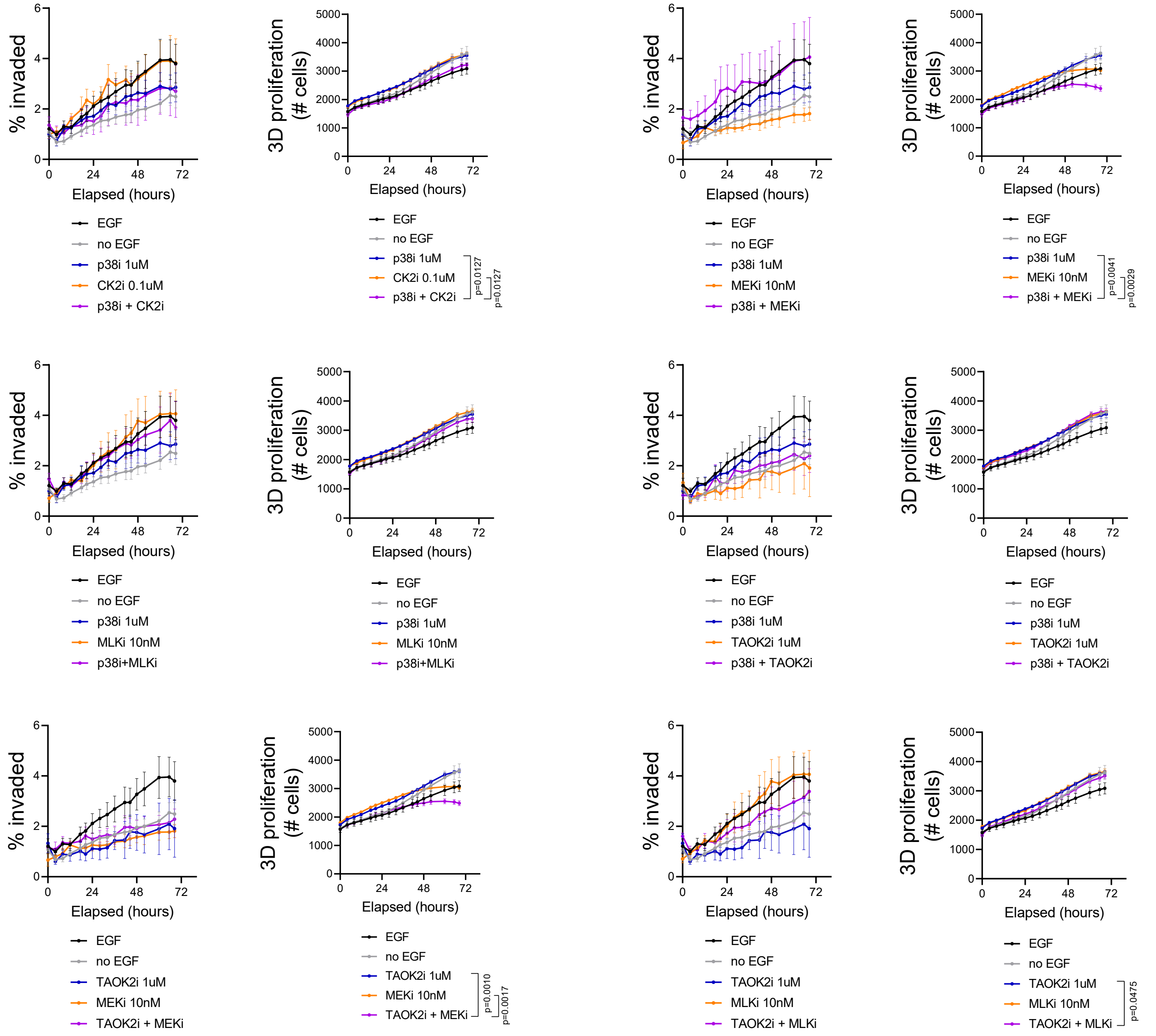

Assay 3 - Dual drug combinations (2)

Conditions tested:
1-EGF (positive control with chemoattractant, but no drug)
2-no-EGF (negative control for baseline invasion, no chemo-attractant, no drug)
3-p38i (0.1µM)
4-MEKi (1nM)
5-MLKi (50nM)
6-TAOK2i (0.1µM)
7-CK2i (10nM)
8-p38i + CK2i
9-p38i + MEKi
10-p38i + MLKi
11-p38i + TAOK2i
12-TAOK2i + CK2i
13-TAOK2i + MEKi
14-TAOK2i + MLKi
15-MEKi + CK2i
16-MEKi + MLKi
17- MLKi + CK2i

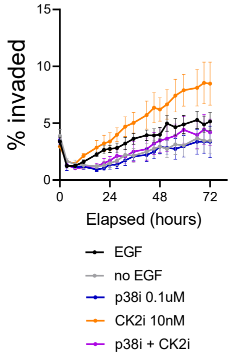

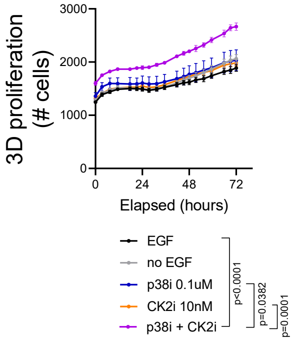

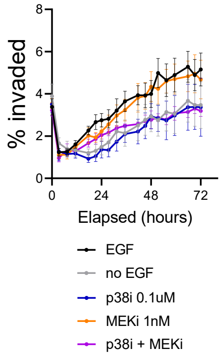

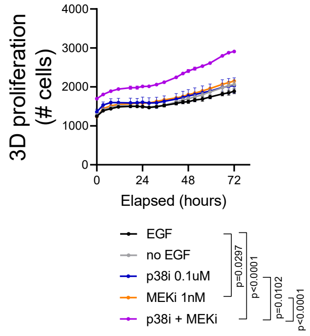

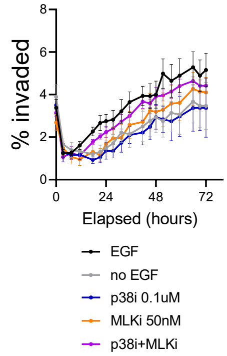

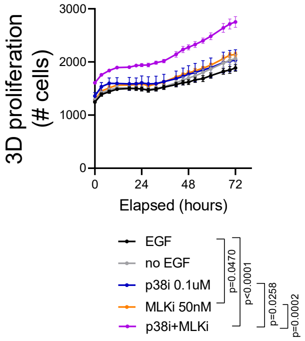

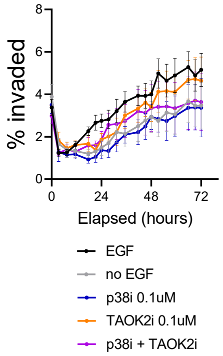

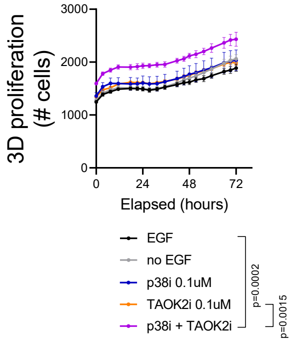

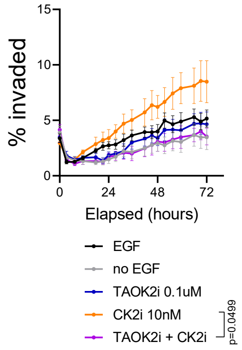

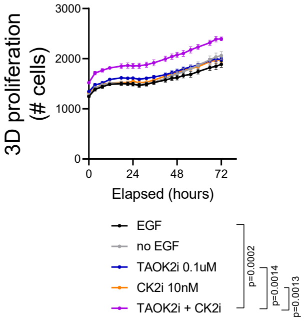

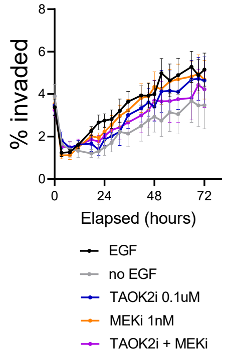

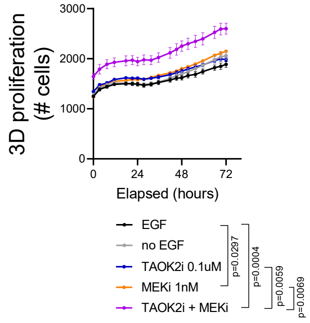

Figure 3 - source data 3 (cont'd)

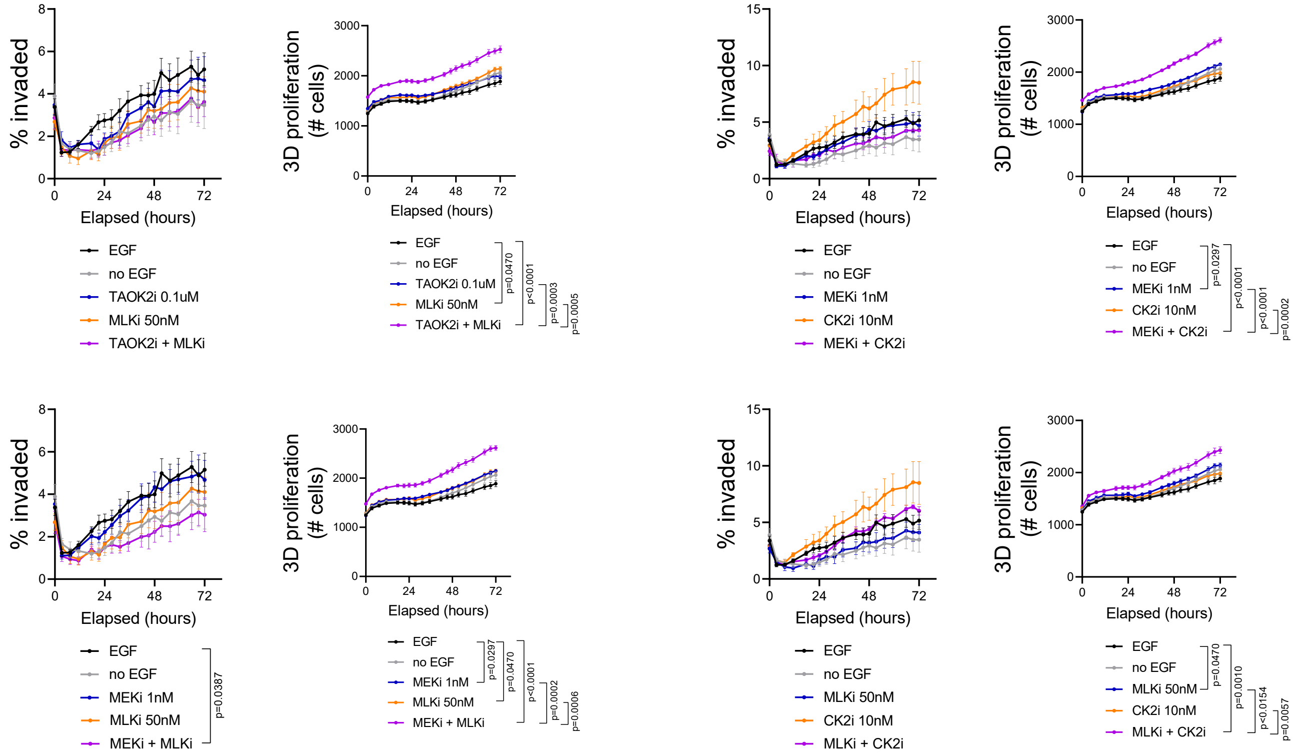

Assay 4 - Dual drug combinations (2) - Repeat

Conditions tested:
1-EGF (positive control with chemoattractant, but no drug)
2-no-EGF (negative control for baseline invasion, no chemo-attractant, no drug)
3-p38i (0.1μM)
4-MEKi (1nM)
5-MLKi (50nM)
6-TAOK2i (0.1μM)
7-CK2i (10nM)
8-p38i + CK2i
9-p38i + MEKi
10-p38i + MLKi
11-p38i + TAOK2i
12-TAOK2i + CK2i
13-TAOK2i + MEKi
14-TAOK2i + MLKi
15-MEKi + CK2i
16-MEKi + MLKi
17- MLKi + CK2i

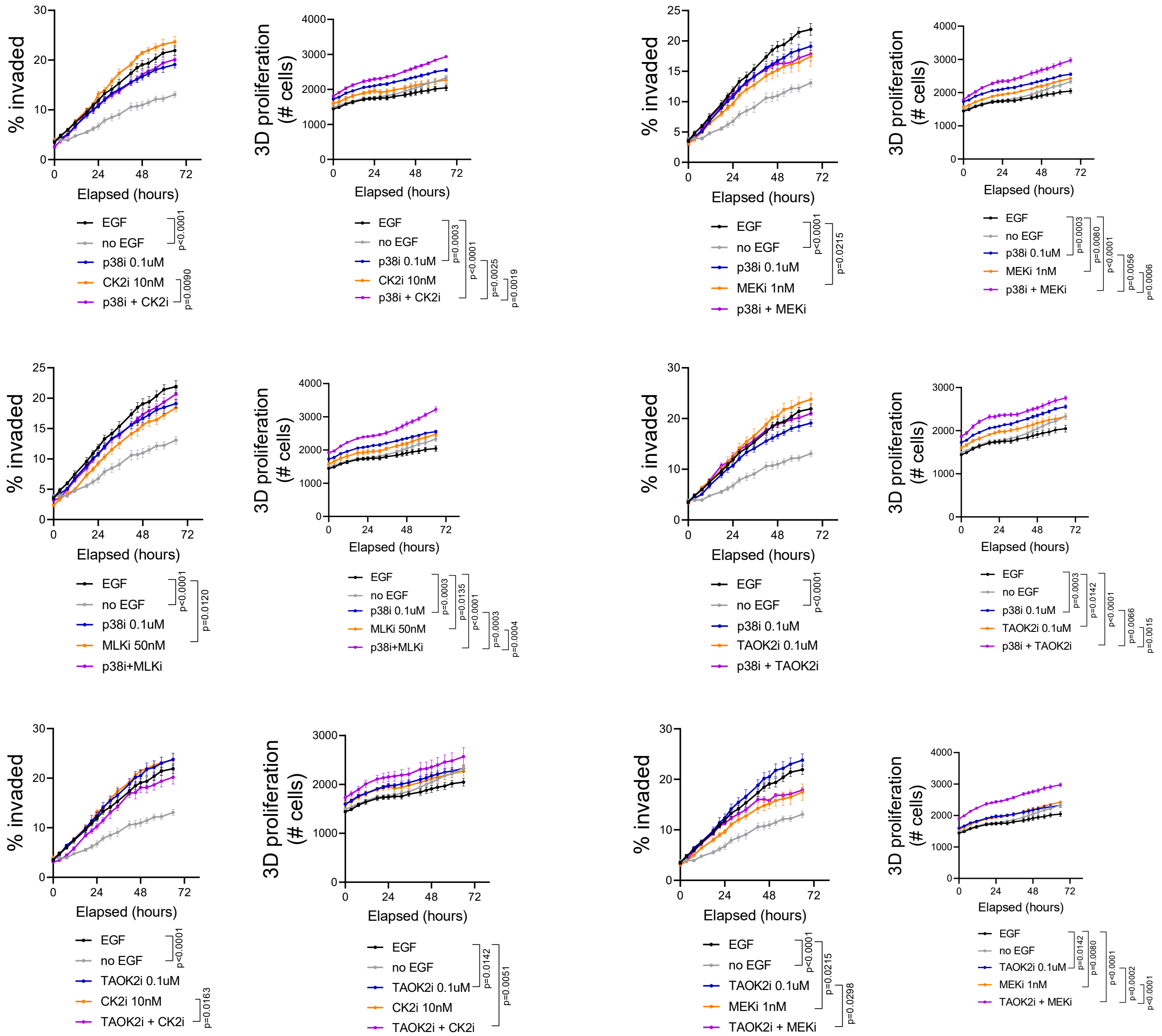

Figure 3 - source data 3 (cont'd)

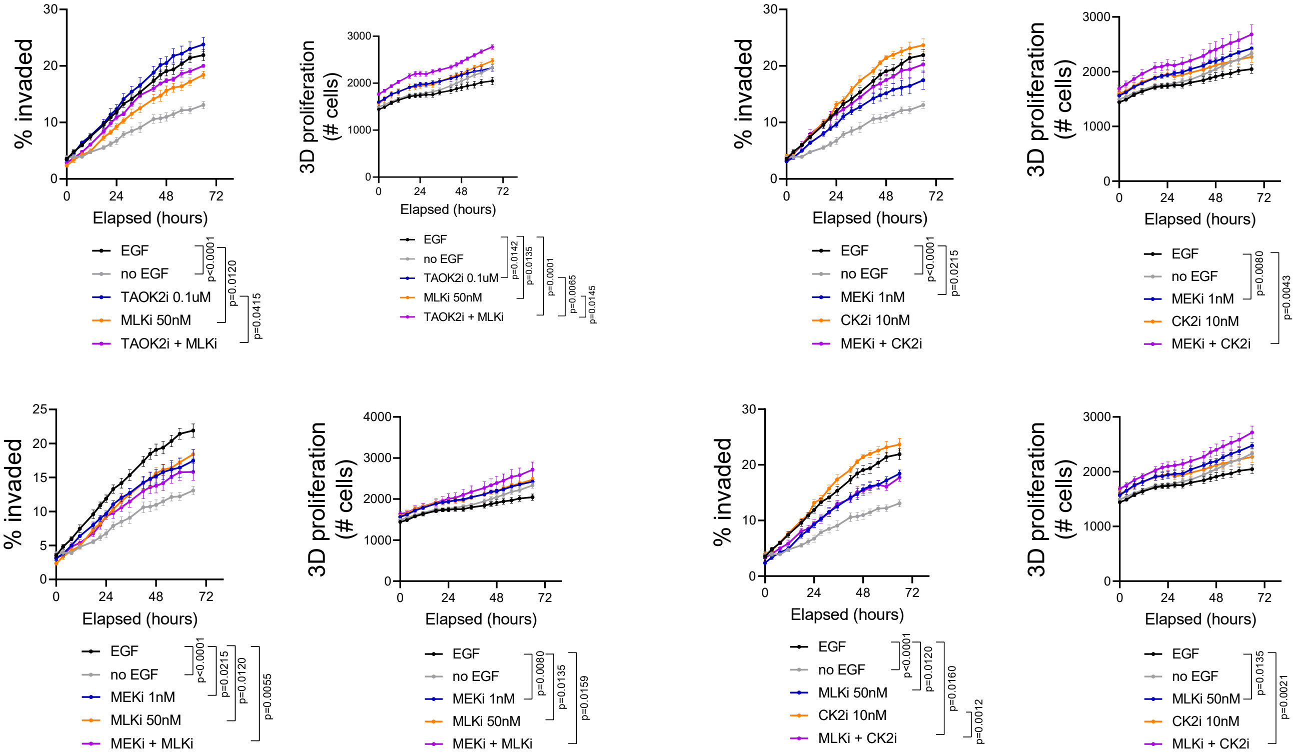

Assay 5 - Multi-drug combinations (1)

Conditions tested:
1-EGF (positive control with chemoattractant, but no drug)
2-no-EGF (negative control for baseline invasion, no chemo-attractant, no drug)
3-MEKi (1nM)
4-MLKi (50nM)
5-TAOK2i (0.1µM)
6-CK2i (10nM)
7-MEKi + MLKi
8-TAOK2i + CK2i
9-MEKi + MLKi + TAOK2i
10-MEKi + MLKi + CK2i
11-TAOK2i + CK2i + MEKi
12-TAOK2i + CK2i + MLKi
13-MEKi + MLKi + TAOK2i + CK2i

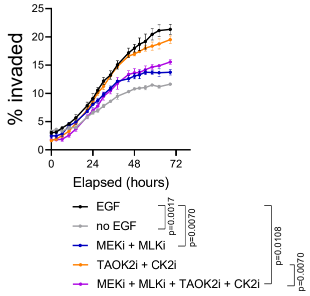

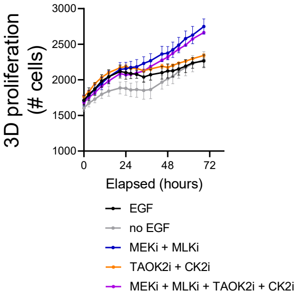

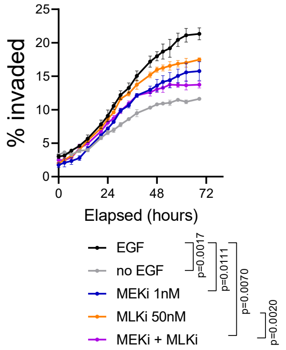

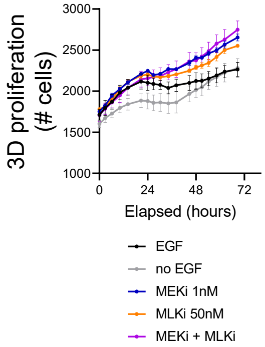

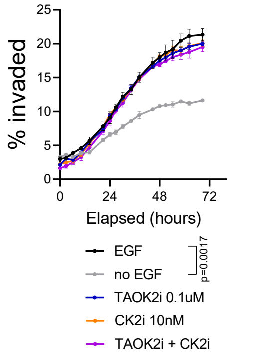

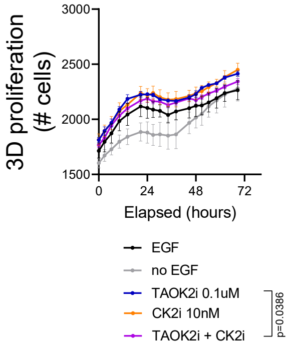

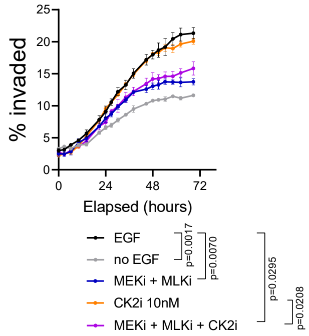

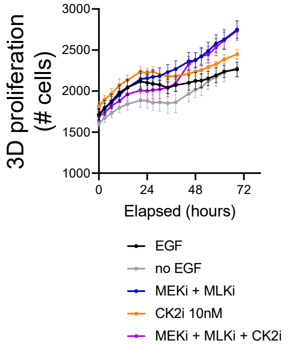

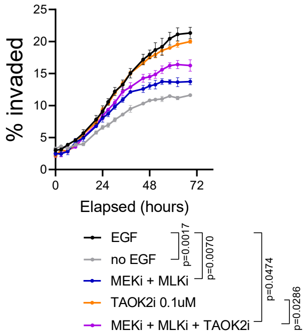

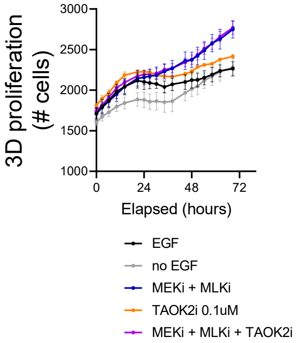

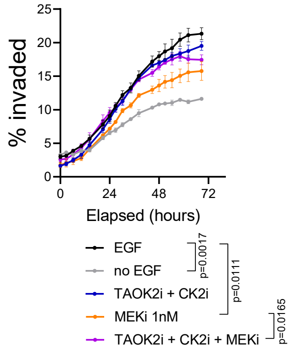

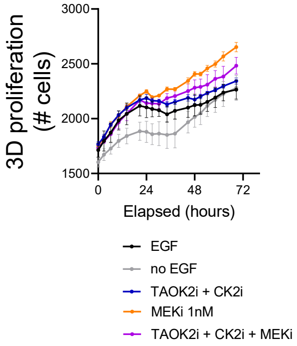

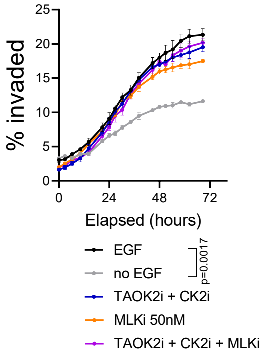

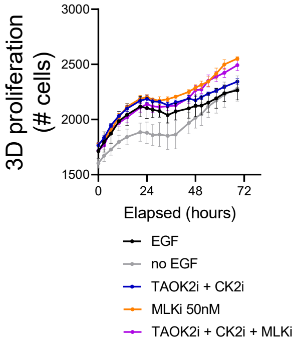

Assay 6 - Multi-drug combinations (2)

Conditions tested:

1-EGF (positive control with chemoattractant, but no drug)
2-no-EGF (negative control for baseline invasion, no chemo-attractant, no drug)
3-p38i (0.1μM)
4-p38i (1μM)
5-JNKi (0.1μM)
6-JNKi (1μM)
7-MEKi (1nM)
8-MLKi (50nM)
9-p38i (0.1μM) + JNKi (0.1μM)
10-p38i (1μM) + JNKi (1μM)
11-MEKi + MLKi
12-MEKi + MLKi + p38i (0.1μM)
13-MEKi + MLKi + p38i (1μM)
14-MEKi + MLKi + JNKi (0.1μM)
15-MEKi + MLKi + JNKi (1μM)

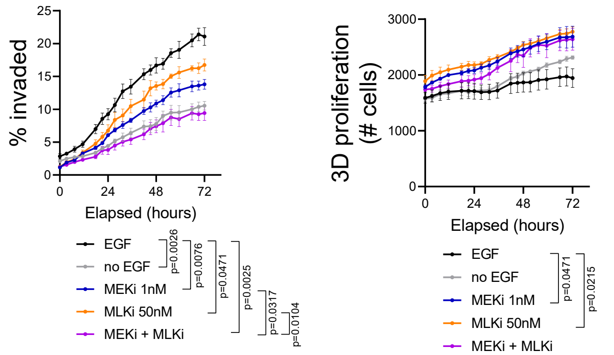

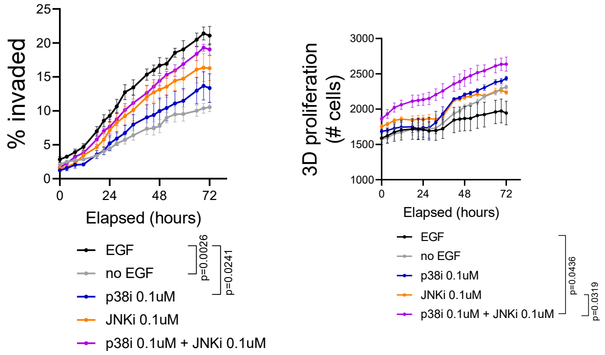

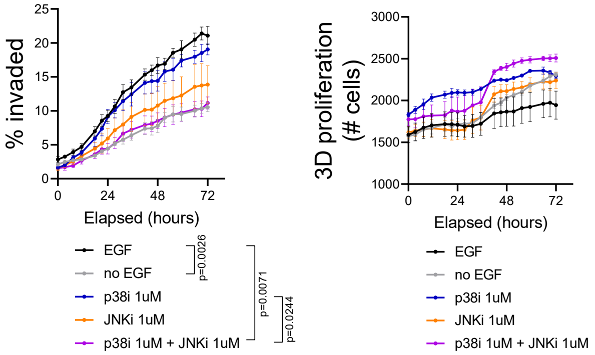

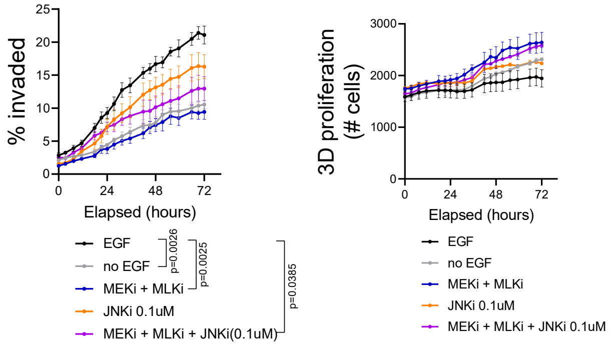

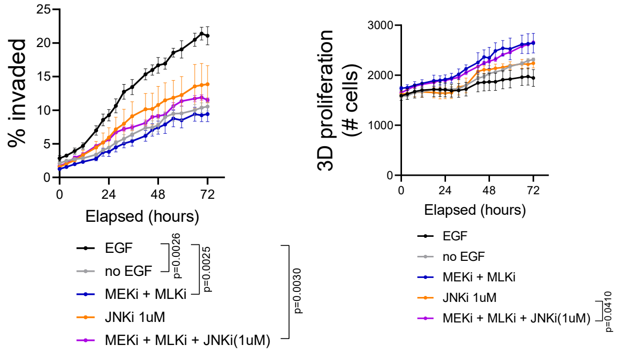

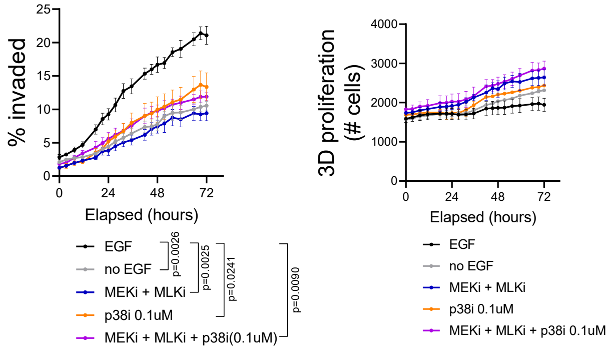

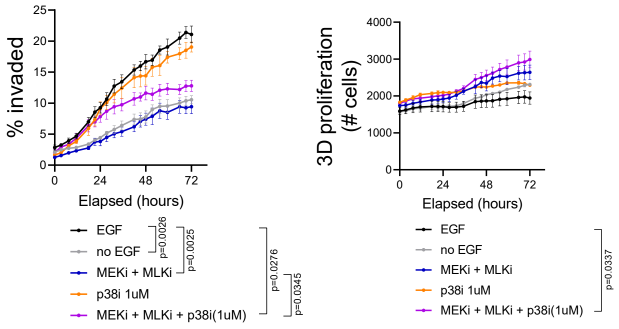

Assay 7 - Multi-drug combinations (3)

Conditions tested:
1-EGF (positive control with chemoattractant, but no drug)
2-no-EGF (negative control for baseline invasion, no chemo-attractant, no drug)
3-p38i (1μM)
4-JNKi (1μM)
5-MEKi (1nM)
6-MLKi (50nM)
7-p38i + JNKi
8-MEKi + MLKi
9-p38i + JNKi + MEKi + MLKi (4DMAPKi)

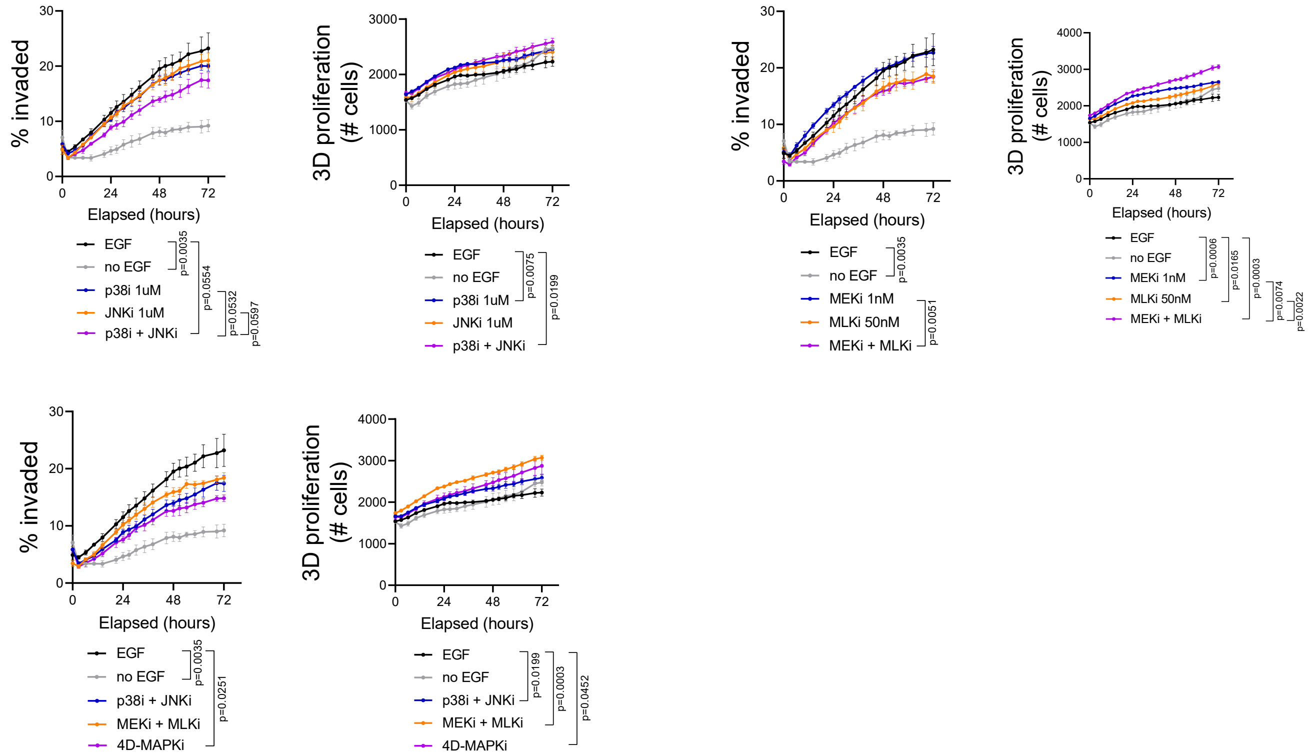

Assay 8 - Multi-drug combinations (4)

Conditions tested:
1-EGF (positive control with chemoattractant, but no drug)
2-no-EGF (negative control for baseline invasion, no chemo-attractant, no drug)
3-p38i (0.1μM) + JNKi (0.1μM) (0.5X)
4-p38i (1μM) + JNKi (1μM) (1X)
5-p38i (2μM) + JNKi (2μM) (2X)
6-MEKi (0.5nM) + MLKi (25nM) (0.5X)
7-MEKi (1nM) + MLKi (50nM) (1X)
8-MEKi (2nM) + MLKi (100nM) (2X)
9-4DMAPKi (0.5X)
10-4DMAPKi (1X)
11-4DMAPKi (2X)

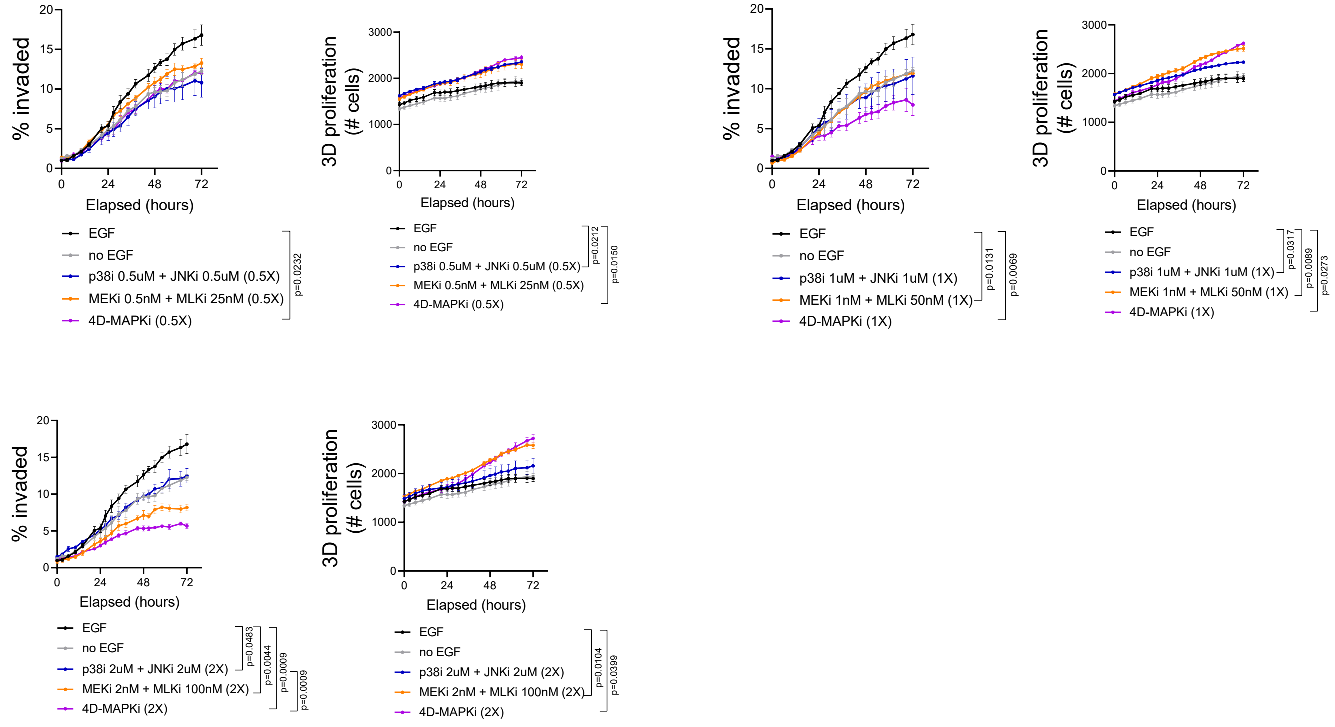

Assay 9 - Multi-drug combinations (5)

Conditions tested:
1-EGF (positive control with chemoattractant, but no drug)
2-no-EGF (negative control for baseline invasion, no chemo-attractant, no drug)
3-p38i (1μM) + JNKi (1μM)
4-MEKi (1nM) + MLKi (50nM)
5-MEKi + MLKi + JNKi (1μM)
6-MEKi + MLKi + JNKi (2μM)
7-MEKi + MLKi + p38i (1μM)
8-MEKi + MLKi + p38i (2μM)
9-p38i + JNKi + MEKi (1nM)
10-p38i + JNKi + MEKi (2nM)
11-p38i + JNKi + MLKi (50nM)
12-p38i + JNKi + MLKi (100nM)

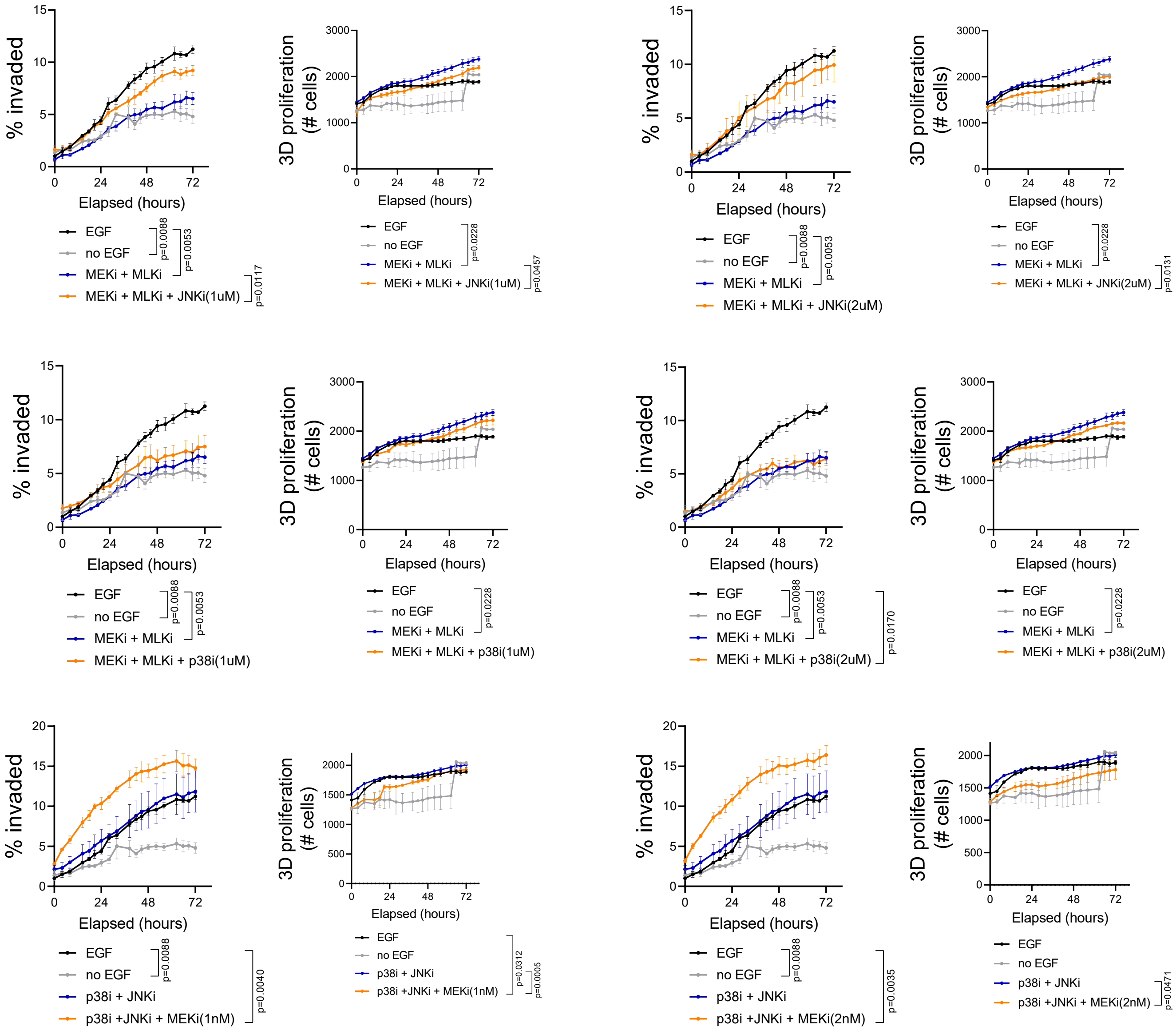

Figure 3 - source data 3 (cont'd)

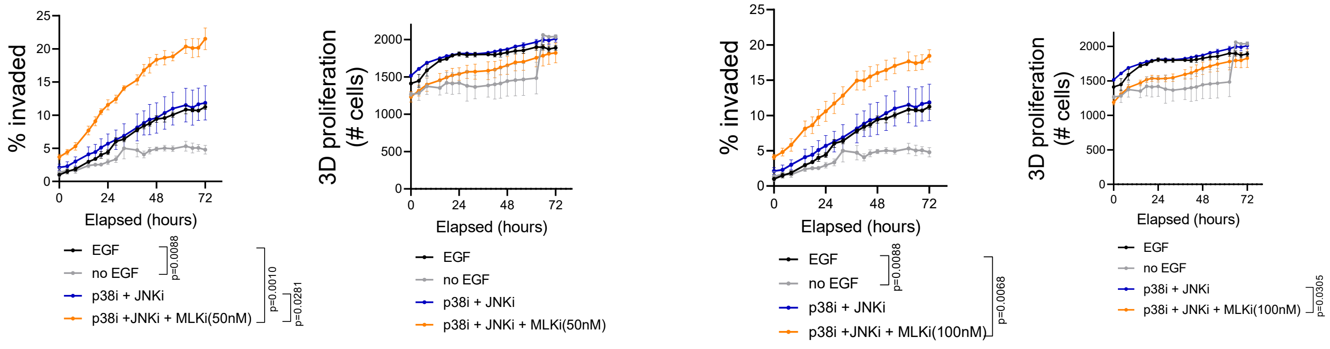

Supplement: Figure 3—source data 2. — Details of individual assays performed to identify potentially effective anti-invasion MAPK inhibitor combinations. Each assay corresponds to an independent 96-well plate designed to test a different set of combinations. Each assay included its own negative (no chemoattractant, no inhibitor) and positive controls (EGF as chemoattractant, no inhibitor). In each graph, the positive control is colored black, the negative control is colored gray, the combination tested is colored purple, and the individual components of the combination alone are colored blue and orange. Statistical significance was determined by a two-way ANOVA test in a pair-wise fashion between each treatment condition and the no-treatment control. [file elife-59696-fig3-data2.pdf]
